# Epstein-Barr virus genetics: talking about the BAC generation

**DOI:** 10.1186/2042-4280-1-6

**Published:** 2010-12-07

**Authors:** Regina Feederle, Emmalene J Bartlett, Henri-Jacques Delecluse

**Affiliations:** 1 German Cancer Research Centre, Im Neuenheimer Feld 242, 69120 Heidelberg, Germany

## Abstract

Genetic mutant organisms pervade all areas of Biology. Early on, herpesviruses (HV) were found to be amenable to genetic analysis using homologous recombination techniques in eukaryotic cells. More recently, HV genomes cloned onto a bacterial artificial chromosome (BAC) have become available. HV BACs can be easily modified in *E.coli *and reintroduced in eukaryotic cells to produce infectious viruses. Mutants derived from HV BACs have been used both to understand the functions of all types of genetic elements present on the virus genome, but also to generate mutants with potentially medically relevant properties such as preventative vaccines. Here we retrace the development of the BAC technology applied to the Epstein-Barr virus (EBV) and review the strategies available for the construction of mutants. We expand on the appropriate controls required for proper use of the EBV BACs, and on the technical hurdles researchers face in working with these recombinants. We then discuss how further technological developments might successfully overcome these difficulties. Finally, we catalog the EBV BAC mutants that are currently available and illustrate their contributions to the field using a few representative examples.

## Introduction

Genetics became an integral part of the Epstein-Barr virus (EBV) research field at an early stage. Identification of viral strains with unusual properties, e.g. incapable of initiating lytic replication, such as Raji, or of transforming B cells, such as P3HR1, later coupled to sequencing allowed the identification of genes or of a group of genes likely to be involved in these functions [1-3]. Although these early EBV mutants appeared spontaneously, they provided an important tool for EBV research. More recently, strategies have been developed to allow researchers to direct mutagenesis of the EBV genome in order to design specific mutants of interest.

The ability to associate specific genes with unique mutant phenotypes was an important step, however, definitive evidence that such phenotypes are associated with specific genes required the construction of revertants. For example, proof that the P3HR1 phenotype was caused by the loss of EBNA2 required the reintroduction of this gene back into the mutant genome through transfection of an EBV DNA fragment that spans the EBNA2 region and the observation that a successfully recombined virus had regained its transforming ability [4,5]. Not only did this observation define EBNA2 as a key transforming gene, it also provided an elegant method to select for recombinants from the background of defective P3HR1 viruses. Indeed, lymphoblastoid cell lines (LCL) generated with supernatants from EBNA-2 transfected P3HR1 cells contained predominantly, if not exclusively, recombinant viruses [4,5]. Therefore, the introduction of EBNA2 provided a potent selection method that could be used to construct mutant viruses. Recombination with a combination of cosmid that contained EBNA2 and of overlapping cosmids that carried a mutated version of another EBV gene, e.g. EBNA3, allowed the generation of EBV mutants that had both re-acquired EBNA2 and incorporated the mutated gene [6]. This technology, based on homologous recombination in eukaryotic cells, has proven invaluable for our understanding of EBV-driven B cell transformation.

A related but distinct strategy for generating EBV mutants consisted of exchanging a viral gene of interest located on the EBV Akata genome with a selection marker such as neomycin [7]. Neomycin resistant Akata cell clones must then be screened to identify those containing successfully recombined mutants. In a further step, mutants often had to be purified from wild type EBV genomes present in the same cell clones. This was usually obtained by inducing the lytic cycle in the clones of interest and subsequently exposing an EBV-negative cell line to the supernatants from these cells. This was performed at a low multiplicity of infection to ensure that every newly infected cell would carry either the mutant or the wild type viruses [7]. The B cell clones would then be screened for the presence of the mutant and selected for phenotypic characterization. This purification step can only be performed if the mutant has retained its ability to lytically replicate and to infect target cells from which they can be expanded. Therefore, mutant viruses that lack the genetic elements essential for either replication or infection cannot, in principle, be obtained by this method. These limitations, combined with the tedious sequential screening steps required by this method, led to the development of a quicker and more versatile strategy for the construction of recombinant viruses [8].

This new method, known as HV BAC technology, was developed in the late 1990 s in several laboratories in Munich for murine cytomegalovirus, EBV, human cytomegalovirus, and murine gammaherpesvirus 68 [9-12]. Since then, several human and animal HV genomes, including herpes simplex virus type 1 [13,26], varicella-zoster virus [14], Kaposi's sarcoma-associated herpesvirus (KSHV) [15,16], rhesus cytomegalovirus [17], rhesus rhadinovirus [18], pseudorabies virus [19], herpesvirus saimiri [20], and Marek's disease virus [21], have been cloned as BACs.

The rationale of the HV BAC approach, which represented an abrupt change of tack from the conventional views of the time, was to clone the complete HV genomes as BACs in order to perform mutagenesis of the viral genome in *E.coli *cells. In a prokaryotic context, eukaryotic genes are not required for persistence of the viral genome and can therefore be extensively modified without any consequences for its maintenance. However, DNA can only persist in bacterial cells if it carries a prokaryotic replicon. Therefore, a BAC flanked by HV-specific sequences was introduced into infected cell lines in order to trigger homologous recombination. This was achieved with great efficiency for alpha-and betaherpesviruses for which fully lytic cellular systems are available, but proved to be a rather arduous task for gammaherpesviruses [10,15]. This might provide an explanation for the fact that only two human KSHV BACs from two different strains have been published [15,16], one of which was obtained with great difficulty by our group, and that only three EBV BACs from two strains have been generated in the last 12 years [10,22,23]. In the same vein, the generation time for EBV mutants is still much longer than for those of alphaherpesvirus mutants.

This review will focus on EBV BAC technology and its mechanics, before highlighting its use as a powerful research tool using specific examples. Therefore, we make no pretense of presenting an exhaustive summary of EBV genetics in general but instead recommend earlier references on that topic [24,25]. We have attempted to catalog all EBV BAC recombinants available to date (Table 1), but apologize in advance to colleagues whose work might have slipped our attention.

**Table 1:** List of available EBV BACs.

gene/locus	protein function	reference
wild-type B95.8		[10,23]

wild-type Akata		[22]

BALF4	virus-cell fusion	[43]

BFLF2	DNA packaging, nuclear egress	[74]

BFRF1	nuclear egress	[75]

BGLF4	protein kinase	[38,76,77]

BGLF5	alkaline exonuclease, virus maturation	[78]

BHRF1	anti-apoptotic	[61]

BHRF1+BARF1	anti-apoptotic	[61]

BLLF1	virus binding	[42]

BMRF1	DNA polymerase processivity	[79,80]

BMRF1+BALF5	DNA replication	[80]

BNRF1	virus transport	[81]

BNLF2a	immune evasion	[37]

BRLF1	lytic replication	[47]

BZLF1	lytic replication	[47]

BZLF1 promoter	lytic replication	[82]

EBNA1	episome maintenance, transactivation	[59]

EBNA2	transformation, transactivation	[83]

EBNA3A conditional EBNA3A	transformation, transactivation	[84] [85]

EBNA3B	unknown	[23]

EBNA3C conditional EBNA3C conditional †	transformation, transactivation	[84] [86]

LMP1	transformation, transactivation	[87]

LMP2A	membrane signal transduction, B cell survival	[88]

oriLyt ZRE sites	lytic replication	[89]

CTCF binding site between oriP and Cp	EBNA2 transcription	[90]

snoRNA1	unknown	[91]

terminal repeats	DNA packaging	[92]

†Akata strain

## Technical issues

### Overview

**Figure 1:**
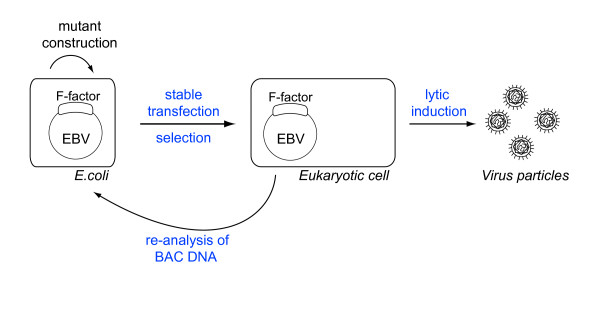
**The EBV BAC system: an overview**. The cloned EBV-BAC can be manipulated in *E.coli *cells using multiple techniques that rely on homologous recombination. The mutated EBV BAC is then introduced into 293 cells and selected with an antibiotic to create a producer cell line from which infectious particles that contain the mutant EBV BAC can be produced. The episomes present in the producer cell line can be extracted and reintroduced in *E.coli *where multiple controls such as restriction analyses, Southern blotting and sequencing, can be readily performed.

The defining feature of EBV BAC technology is the ability to shuttle the recombinant viruses between prokaryotic and eukaryotic backgrounds (Figure 1). As a plasmid in *E.coli*, the EBV BACs can be easily modified using the highly versatile genetic tools developed in these cells. Foreign sequences can be added to the recombinant viruses, as long as they stay within the constraints imposed by the limits of the EBV capsid packaging capacity. Examples are selection markers such as antibiotic resistance cassettes, genes encoding fluorescent proteins, or tumor antigens. When using BAC technology, extensive controls can be performed (see below), including the possibility to generate revertants of the mutated EBV BACs. All these techniques are very powerful and not more complex than conventional Molecular Biology cloning techniques.

The mutated EBV BAC is also a genuine virus, provided it is transferred back to a eukaryotic environment in which the recombinant viral DNA can be packaged into infectious virions. This obviously requires introduction of the EBV BAC DNA into cells that support lytic replication. Furthermore, lytic replication must be easily initiated in these cells, if possible in a physiological way, e.g. through expression of the trans-activators BZLF1 and BRLF1. There are only a limited number of cell lines that fulfil these conditions. Furthermore, we have observed on many occasions that only a subset of clones generated from a cell line transfected with the same EBV BAC will sustain replication to a useful level. This probably reflects the marked propensity of gammaherpesviruses to maintain tightly latent infection, at least *in vitro*. This characteristic again contrasts with the relative ease with which alpha-and betaherpesvirus BACs undergo replication following transfection of the recombinant viral DNA into permissive cells [see for example [11,13,26]]. Indeed, transfected alpha-HV genomes will spontaneously initiate lytic replication and launch a first round of virus production from which the infection can be propagated to neighboring cells. Thus, the difficulty associated with the generation of high-quality producer cell lines is the current bottleneck of EBV BAC technology with regards to gamma-HV applications. To add insult to injury, some of these gamma-HV producer cell lines tend to lose their ability to support lytic replication upon induction with time. The biological mechanism behind this phenomenon is unknown to us, but it necessitates careful freezing of multiple aliquots of the cell lines at an early time point. Despite these limitations, we have never lost any producer cell line, and go back to early passage freezing as soon as the replication rates decline.

### Available systems

Three recombinant wild-type EBVs have been constructed to date (Table 1) [10,22,23]. All of these were constructed by insertion of the prokaryotic F-plasmid, or F-factor, in either the B95.8 [10,23] or the Akata strain [22]. The two B95.8 BACs differ in the site of the F-plasmid insertion, either at the site of the B95.8 deletion [10], or in the major internal repeat region [23]. In the Akata BAC, the F-plasmid is inserted in the BXLF1 open reading frame that encodes the viral thymidine kinase and was previously shown to be dispensable *in vitro *[22]. The insertion site of the F-plasmid does not affect the phenotype of the virus [10]. In all three constructs, eukaryotic and prokaryotic resistance cassettes were inserted into the F-plasmid (hygromcin, neomycin or puromycin and chloramphenicol or kanamycin, respectively). The Akata BAC also contains a unique I-PpoI restriction site, flanked by a SV40 enhancer/promoter and a polyA site which allows conventional cloning of genes to be expressed at high levels on the viral recombinant [22]. It is interesting to note that all three BACs were introduced into different cell lines. The Akata BAC was re-introduced into EBV genome-negative Akata cells, one of the B95.8 BACs was introduced into 293T cells [23] and the other into HEK293 or AGS cells [10,27]. It is therefore possible to generate recombinant EBVs in B cells or in different epithelial cells. B95.8 virus production was initiated either by introducing BZLF1 in the producer cell lines, alone or in combination with BRLF1, or phorbol 12-myristate 13-acetate and n-butyrate. BZLF1 can be directly transfected into 293 cells or delivered via infection with an adenovirus vector. The Akata BAC virus can be induced by crosslinking of surface immunoglobulins, as initially developed for Akata cells [28].

### Mutant generation

**Figure 2:**
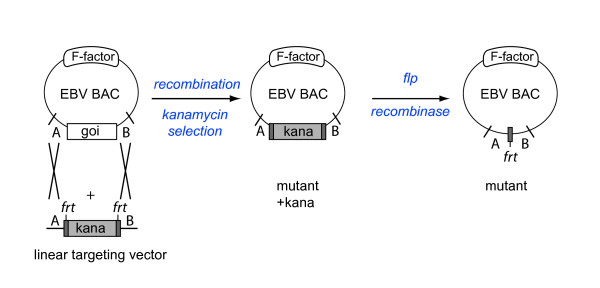
**EBV BAC mutagenesis in *E. coli***. Recombination with linearized targeting vectors. This method allows deletions or exchanges of genetic material from the EBV DNA against foreign sequences. The latter can be mutated versions of an EBV gene, or DNA fragments of cellular or bacterial origin. Selection of successfully recombined BACs requires the introduction of an antibiotic resistance cassette flanked by Flp-recombinase target (FRT) sites. Transient introduction of the FLP recombinase allows excision of the antibiotic resistance cassette.

**Figure 3:**
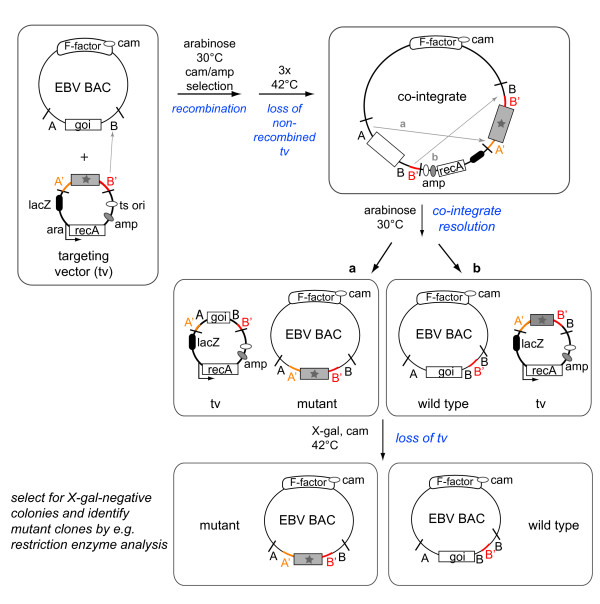
**Chromosomal building in *E. coli***. Recombination with circular targeting vectors. Chromosomal building is one of several techniques that allow seamless mutagenesis. It is based on a targeting vector that carries: i) an antibiotic resistance cassette e.g. ampicillin (amp); ii) the sequence to be introduced into the EBV-BAC (represented by the grey shading and star) flanked by EBV-specific sequences (designated as A and B on the EBV BAC and A' and B' on the targeting vector) that will determine its site of insertion; iii) the gene that encodes the lacZ enzyme; and iv) a temperature-sensitive origin of replication that is operative only at 30°C. The targeting vector is introduced into *E.coli *cells that carry the EBV BAC. Recombination between both prokaryotic episomes is performed by a recA recombinase present on the targeting vector, whose expression is driven by an arabinose-inducible promoter. Homologous recombination can be initiated anywhere within the regions of homology (indicated by an arrow). The antibiotic resistance cassettes present on the targeting vector (amp) and on the EBV BAC (cam) allow the selection of co-integrates, which are a fusion vector comprising the targeting vector and the EBV BAC. Propagation at 42°C (non-permissive temperature) forces the loss of free targeting vectors. A second round of recombination resolves the co-integrates and reconstitutes both the EBV BAC and the targeting vector. Depending on which flanking region initiates resolution of the co-integrate, a recombinant EBV BAC containing either the foreign sequence (a) or the wild type (b) will be generated. Reconstituted targeting vectors are eliminated by culture at non-permissive temperature. Candidate clones are assessed for their sensitivity to ampicillin and expression of the lacZ gene. LacZ-negative and ampicillin-sensitive clones, indicative of reconstituted EBV BACs, are then submitted to restriction enzyme analysis, colony PCR or any other appropriate technique. goi: gene of interest.

Two methods are mainly used to construct mutants of the EBV BACs, both are based on homologous recombination between wild type and mutant versions of a gene of interest [29] (Figure 2 and 3). Both methods require the genetic elements to be exchanged to be flanked by identical DNA sequences in order to initiate recombination. Thus the targeting vector consists of the mutated gene flanked by sequences homologous to the viral genome. If ablation of a genetic element is required, its flanking regions are simply juxtaposed.

One method makes use of linearized targeting vectors to initiate recombination (Figure 2). In this case, as a consequence, the wild type target gene will be excised from the EBV BAC and the mutated versions of the gene of interest inserted in its position. However, this event is relatively rare, and strict selection methods must be applied to successfully identify the properly recombined EBV BACs. To this aim, an antibiotic resistance cassette is inserted next to the mutated gene. As a result, recombination not only exchanges the wild type gene against its mutated version but also inserts the antibiotic resistance cassette. This phenotypic marker can be flanked by Flp-recombinase target (frt) sites. Transient transfection of the Flp recombinase into cells that contain the EBV BAC then allows excision of the antibiotic resistance cassette, leaving behind the mutated gene and one Frt site.

Another method, dubbed 'chromosomal building', uses circular targeting vectors carrying multiple selection markers, an arabinose-inducible recA gene, and the mutated version of the gene of interest, flanked by two regions of homology that will determine the site of homologous recombination (Figure 3). The selection markers typically include a temperature-sensitive origin of replication, an antibiotic resistance gene such as ampicillin, and the lacZ gene. Upon transcription of RecA, recombination is initiated and leads to fusion of the targeting vector with the EBV BAC via one of the regions of homology. This yields a co-integrate that carries both the wild type and the mutated sequence, both flanked by identical sequences from the EBV genome. The co-integrate therefore carries two identical sets of the flanking regions of homology. If the chloramphenicol resistance gene is present on the BAC then the co-integrate can be selected for by growing the bacterial cells in the presence of chloramphenicol and ampicillin, which is present in the target vector. Shifting the cells to a non-permissive temperature will eliminate all non-recombined targeting plasmids after a few cell divisions due to the presence of the temperature-sensitive origin of replication. Co-integrate formation can be easily monitored by restriction enzyme digest and is usually very efficient. Once co-integrate formation is confirmed, cells are then shifted back to arabinose-containing medium at a permissive temperature to induce recombination within the co-integrate. The EBV BAC and the targeting vector can initiate recombination through the region of homology located to the right or left of the gene of interest and its mutated version in order to form a co-integrate. Reciprocally, this co-integrate can then be resolved through recombination of either of the homologous flanking sequences, resulting in the potential for two alternative plasmids to be generated during this process. If resolution of the co-integrate takes place through the flanking region engaged in the generation of the co-integrate, the mutated gene remains on the targeting vector, and the EBV BAC wild type sequence is reconstituted. In contrast, if resolution occurs through recombination of the flanking regions not used for construction of the co-integrate, the targeting vector is recombined with the wild type copy of the gene and the mutated EBV BAC is generated. Finally, in order to induce the removal of the targeting vector from the bacterial cells, cells are propagated at non-permissive temperature. Clones can then be assessed for the loss of lacZ and sensitivity to ampicillin which is indicative of successful elimination of the targeting vector. Candidate clones require screening by restriction enzyme analysis, colony PCR or any other appropriate technique.

The enzymes typically used for recombination are either *E.coli *RecA or λ-phage Red recombinase, used alone or in combination with RecE and RecT from the Rac prophage [30,31]. Both are very potent recombinases and generation of co-integrates is usually straightforward. However, resolution of co-integrates does not yield an equal percentage of wild type and mutant genomes. Instead, the majority of resolved co-integrates will be revertant wild type clones (from 51 to 98% in our experience). More recently, positive/negative selection methods using the *galK *gene or a combination of two selection markers, such as kanamycin and streptomycin, have also been reported [32,33]. A very efficient alternative positive/negative selection method combines Red recombination and endonuclease I-SceI cleavage. In this strategy, the positive selection marker that was used to introduce the target modification is removed by the combination of I-SceI cleavage and Red recombination through sequence duplications that were previously introduced into the targeting vector [34].

Each method has advantages and disadvantages depending on the potential applications of the EBV BAC mutants. The linear targeting vectors can be designed quickly and construction of the mutants usually takes only a few days. However, even after elimination of the antibiotic resistance cassette, prokaryotic sequences will usually be left behind. Most of the time, these foreign sequences have no influence on viral gene expression and they can even be advantageous in the case of mutants that carry a complete deletion of a given gene as they keep the total size of the virus constant. However, if more subtle mutations are required, circular targeting vectors, *galK *selection or two-step Red recombination/I-SceI cleavage would be the methods of choice.

Random transposon mutagenesis has been used to generate libraries of CMV or PrV mutants [19,35]. The relative inefficiency with which EBV BACs can be packaged into infectious viruses and selected for a particular phenotypic trait among a complete mutant library renders this approach perhaps less attractive for EBV.

### Revertant generation

By definition, a revertant is the reversion of a mutant virus to the wild type configuration. Revertant viruses are often used as controls to demonstrate that the phenotypes of mutant viruses can be attributed to a specific mutation or gene deletion, and not to any secondary mutations that may have occurred elsewhere in the genome during mutagenesis. Subsequently, a revertant should be absolutely identical to the recombinant wild type sequence and must not carry any foreign sequences. Therefore, typically, the chromosomal building technique, *galK *selection or Red recombination coupled to I-SceI cleavage will be used for generating revertants. The method for generating revertants is identical to the one previously described, except that in this case the mutant is used as the reference genome and the wild type sequence is introduced into the targeting vector. Alternatively, allelic exchange following conjugation between bacterial cells that contain the mutated HV-BAC and other bacterial cells that contain the wild type allele cloned onto a vector that permits conjugation has also been successfully used to construct revertants [19].

### Producer cell lines

Once the mutant and the revertant genomes have been obtained, they can be stably introduced, using various methods, into the cell line to be used as a producer cell line. This is most commonly achieved by direct transfection or co-culture between the bacterial cells that carry the EBV BAC and the eukaryotic cells to be transfected [23]. After selection with an antibiotic that is toxic to EBV BAC-negative eukaryotic cells, resistant clones are then tested for their ability to support the lytic cycle. We select clones that carry intact EBV BAC episomes and that produce viral titers in excess of 10^7 ^genome equivalents per ml supernatant, as assessed by a qPCR-based method (please refer to the control section below for more detail).

### Controls

**Figure 4:**
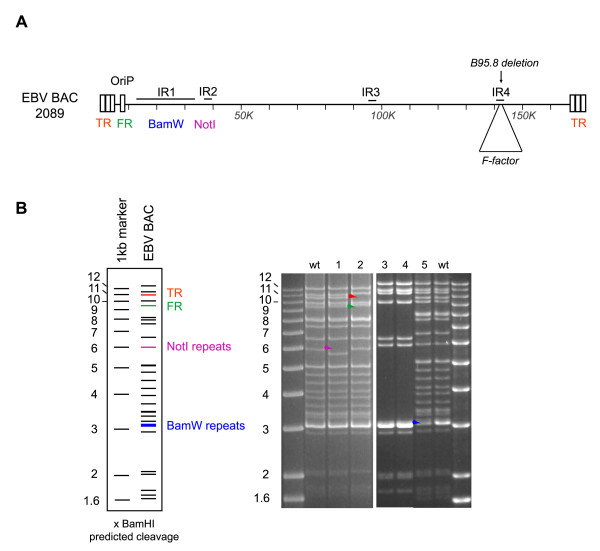
**Assessing the EBV BAC structure by restriction enzyme analysis**. (A) Schematic overview of the EBV genome indicating the position of the various DNA repeats. IR: internal repeats, TR: terminal repeats, FR: family of repeats. (B) Restriction analysis of EBV BACs stably transfected into 293 cells. The left panel shows the predicted position of viral fragments after BamHI restriction. The right panel shows actual examples of abnormal EBV BACs rescued from producer cell lines. One EBV BAC carried fewer NotI repeats than the control (lane 1, purple arrow), another EBV BAC contained fewer TRs but more FRs (lane 2 red and green arrows, respectively), and a third recombinant carried fewer BamHI-W repeats (lane 5, blue arrow). Examples of large deletions (lane 3 and 4) are also shown. All of these abnormal clones were discarded. wt: wild type.

With the increasing use of EBV recombinants, we feel that it is important to expand on the issue of appropriate controls. Passaging of the EBV genome and introduction of mutations via homologous recombination can be accompanied by multiple unintended secondary mutations. This can include gross rearrangements such as a reduction in the number of repeats (BamHI-W repeats, terminal repeats, NotI repeats, etc.) or massive deletions in the viral genome that can be easily detected by restriction enzyme analysis of EBV recombinant plasmid preparations, but can also include point mutations that can be more difficult to identify (Figure 4). Therefore, it is important to assess the structure of EBV recombinants not only during construction of the mutant, but also after establishment of the producer cell line. To this aim, EBV episomes present in the producer cell line can be extracted and re-introduced in *E.coli *to be re-analyzed by restriction enzyme analysis. As EBV producer cell lines are monoclonal in origin and carry on average 5 to 10 episomes, analysis of 5 to 10 bacterial clones from each putative EBV producer cell line will give a good overview of the quality of the producer cell line. Altogether, we find that up to two-thirds of producer cell lines carry a mixture of intact and defective EBV genomes (Figure 4).

Alpha-and betaherpesvirologists have been using revertants and trans-complementation as controls for decades. Revertants use the mutant genomes as a basis to reconstitute the wild type sequence to ensure that no additional mutations were introduced during mutagenesis. Indeed, if a mutant were to carry crippling mutations in addition to the mutation of interest, the revertant will not recover all wild type properties. Similarly, trans-complementation consists of transient or stable introduction of an expression plasmid encoding the genetic element previously deleted and in most cases it is a better control than a revertant. Indeed, viral gene loci frequently carry multiple genes that partially overlap and inactivation of one gene might disrupt expression of other genes in immediate proximity. Whilst in that case a revertant will correct the phenotype and therefore overlook the mutant's construction flaws, trans-complementation will not. In principle, perfect trans-complementation, i.e. complete reversion of the mutant's phenotypic traits upon reintroduction of the missing genetic element, renders the construction of a revertant dispensable. However, there are many cases where trans-complementation is not possible; deletion of a cis-element such as an origin of replication obviously cannot be complemented. In addition, some cells, e.g. primary B cells or LCLs cannot be efficiently targeted by trans-complementation. EBV-derived vectors that can be replicated and packaged or lentiviruses have been previously used, but these also target B cells with limited efficiently.

Several strategies have been developed in an attempt to circumvent these limitations. Complementation vectors that carry a drug-resistance gene and therefore allow selection of the complemented cells can be used. Alternatively, LCLs can be transfected with complementation plasmids that also encode a truncated nerve growth factor receptor (NGFR). Transfected cells can then be purified using NGFR-specific antibodies [36,37]. Another possibility is to target B cells with a retrovirus or an expression plasmid that encodes a fluorescent protein in addition to the gene used for complementation. This would enable the transfected cells to be FACS-sorted. Issues regarding the level of expression and timing of trans-complementation compared to wild type gene expression provide an additional layer of complexity. To say that gene regulation of the viral genome or of an expression plasmid frequently differ is stating the obvious. We have previously encountered this problem when working with viral enzymes whose powerful effects require finely tuned expression both in intensity and timing [38]. The use of conditional systems (e.g. tetracycline-inducible promoters) might offer a more versatile solution to these problems [39]. In all of these cases, revertants become indispensable as controls.

Recent technological developments might change this view. The availability of high-throughput sequencing platforms in a growing number of research centers renders it now possible to obtain the complete viral genome sequence for large viruses such as EBV. Therefore, the presence of adventitious mutations in mutants could, in principle, be excluded. However, we have used this technology to sequence purified EBV BACs and found that sequencing of GC-rich sequences, in particular within the repeats that abound in the EBV genome, is difficult (unpublished data). As a result, seamless assembly of the complete sequence was not possible and we could not exclude the presence of small deletions or unintended rearrangements. In addition, deep sequencing typically results in multiple reads of the same DNA segment, some of which will carry point mutations. Distinguishing sequencing mistakes from genuine mutations present in only a subset of the sequenced EBV BAC molecules also proved impossible. Therefore, we feel that the construction of revertants will continue to be an important control.

Tiling arrays that consist of oligonucleotides spanning the entire BAC sequence have been used to detect mutations in HV BACs. In this case, hybridization of wild type viral DNA is used as a reference sample and allows direct comparison with BAC DNA, as recently shown for rhesus rhadinovirus BAC [18].

One frequently heard criticism about the use of BAC-based mutants, as compared to mutants constructed in eukaryotic cells, is that the transfected DNA has a different methylation pattern. Indeed, HV BACs carry a bacterial epigenetic signature, whereas mutant viruses constructed in eukaryotic cells obviously maintain a eukaryotic methylation pattern. However, for generation of mutants in eukaryotic systems, new rounds of infection are required and it has been shown that EBV DNA in infectious particles is unmethylated. Therefore, cells to be used as producer cell lines become infected with unmethylated genomes [40,41]. The only potential problem of EBV BACs is therefore that they initially carry bacterial-type methylation residues. However, these will be lost after a few cell divisions and are unlikely to adversely interfere with viral functions. The observation that alpha-and betaherpesvirus BACs efficiently initiate virus production after transfection into a permissive cell line certainly supports this view [11,13,26]. Another important aspect, which was alluded to in the previous section and which may also be related to methylation patterns, is the ability of a cell clone to support the EBV life cycle. As already mentioned, only a minority of EBV BAC-containing clones will produce high titers (i.e. as high as marmoset LCLs such as B95.8). However, many of these will initiate replication and progress into the lytic replication phase up to a variable stage but will not complete it. Does abortive lytic replication stem from a defect limited to the late stages of lytic replication, or will this defect affect replication altogether? In the first case, such a producer cell line would probably be valid for the study of early replication events, however, in the second case it runs the risk of delivering artefactual results. There is currently no experimental evidence to distinguish between these alternatives, however, given these possibilities we feel that it is probably safer to restrict studies to producer clones that generate virus titers in the range of 10^7 ^genome equivalents/ml upon induction. In the case of viruses in which a mutation that impairs replication has been purposefully introduced, these titers should be achieved after complementation with the deleted genetic element.

## Applications

### EBV Infection

The genetic analysis of EBV functions required for viral infection has mainly been performed with mutants generated by conventional construction methods. Two published studies made use of EBV BAC mutants in which either the BLLF1 or BALF4 gene, coding for gp350 or gp110, respectively, were deleted [42,43]. A virus that lacks gp350 infects primary B cells less efficiently than its wild type counterparts, but the virus nevertheless remains infectious. Gp350 was thought to function primarily function in B cell binding. However, gp350 mutant viruses maintain their ability to bind to B cells, although less efficiently, relative to controls, suggesting that additional viral ligands may contribute to B cell binding. To determine whether gp350's functions are restricted to binding, we compared infection rates between ΔBLLF1 viruses that had or had not been complemented with an antibody chimera that comprises the gp350 transmembrane domain and an antibody directed against CD21, EBV's main receptor on B cells [44]. Whilst ΔBLLF1 viruses that expressed an antibody against CD21 at their surface bound to B cells as efficiently as ΔBLLF1 complemented with the entire gp350 protein, they were not as efficient in infecting B cells [44]. We concluded that gp350 serves additional functions than merely binding to its target cells.

### EBV Replication

EBV replication requires sequential steps of viral protein synthesis. The immediate early proteins that initiate this process are transactivators that stimulate the synthesis of early and late proteins involved in DNA replication and construction of the infectious viruses. Two transactivators, Zta and Rta, encoded by BZLF1 and BRLF1 respectively, have been shown to initiate lytic replication [45,46]. Studies using mutants that lack either Zta or Rta showed that both proteins are required for virus production [47]. Their functions are therefore not redundant; Zta and Rta were found to preferentially activate different early and late proteins. Furthermore, the 293/ΔBZLF1 producer cell line has proven to be very useful for a detailed genetic analysis of BZLF1 functions [48-56]. Indeed, this virus producer cell line can be efficiently transfected and the endogenous BZLF1 gene does not interfere with transfected Zta mutant proteins. More generally, the 293/ΔBZLF1 producer cell line has been used as a completely replication-negative EBV infected cell line and LCLs immortalized with ΔBZLF1 viruses provide a helpful control in the analysis of T cells directed against lytic proteins [37,57,58].

### EBV-mediated transformation

The EBV latent genes have been extensively studied using overlapping cosmid technology. However, EBNA1 has not been the focus of these types of investigations. One reason for that is that EBNA1 is required for EBV maintenance in latently infected B cells. BAC technology allowed the construction of a 293 cell line expressing EBNA1 *in trans*, into which the EBNA1-negative mutant can be transfected [59]. The EBNA1 mutant virus proved to be 10^4 ^times less infectious than its wild type counterparts. Indeed, EBV could persist in B cells only through integration of the viral DNA within the cellular genome, provided that the integration did not impede latent gene protein synthesis. Furthermore, the 293/ΔEBNA1 producer cell line was useful for investigating the role of EBNA1 as a transactivator of other latent proteins [60].

Although the active latency phase of EBV infection is classically thought to be mediated by the latent genes, B cells exposed to viruses devoid of BALF1 and BHRF1 died of apoptosis immediately after infection [61]. Therefore, the concept of latent genes, or rather of viral genes serving dual lytic and latent functions could be extended to these two viral bcl2 homologs. Importantly, viruses that lacked only one of these genes were indistinguishable from wild type viruses, suggesting either that BALF1 and BHRF1 interfere with the cell apoptosis programme in two different ways, or that a high expression level of anti-apoptotic proteins is required to counteract cell death [61].

### Immune evasion

In the last five years, a number of viral proteins were found to block immune recognition of viral proteins during lytic replication (BGLF5, BZLF2, BILF1, BNLF2a) [37,58,62-64]. The direct contribution of BNLF2a in immune evasion was proven using a EBV BAC devoid of the BNLF2a gene [37]. This recombinant virus elicits a stronger MHC class I T cell response against viral lytic genes than wild type viruses.

### VLPs as a source of viral antigen

Virus-like particles (VLP) have been successfully used as preventative vaccines against Hepatitis B viruses or Papillomaviruses [65,66]. Supernatants from induced EBV producer lines also contain defective virions including VLP that lack viral DNA and light particles (LP) that lack both viral DNA and capsids. These abnormal infectious particles also represent minor sub-populations in supernatants from cultures infected with HSV or CMV [67,68]. We previously reported the phenotypic traits of an EBV mutant devoid of terminal repeats (TR) that produces large amounts of VLP and LP, but no intact virions. Supernatants from induced 293/ΔTR producer cells were found to elicit a potent CD4+ cytotoxic T cell response against various components of the mature virions [69,70]. EBV VLP could therefore be used as a source of antigens in T cell therapy protocols or even as a preventative vaccine. Whether the immune response elicited by VLP/LP would be sufficient to afford protective immunity against wild type virus infection *in vivo *cannot be determined using the data currently available.

## Future directions

Although there are multiple ways in which the BAC system could be improved, some areas appear to be in particular need of improvement. In contrast to alpha and beta HV, the number of cloned EBV strains is restricted to only B95.8 and Akata. The reason for this state of affairs is obvious; the BAC system requires successful recombination in eukaryotic cells, a process with low efficiency. In addition, introduction of the F-plasmid in EBV-positive cell lines is sometimes difficult, particularly in human LCLs. As establishment of LCLs is the easiest way to expand EBV, the number of viral strains amenable to cloning is restricted. Nevertheless, we feel that the availability of more EBV BACs would substantially increase the power of this technology.

Several HV BACs have been improved to include a mechanism that enables removal of the BAC from the recombinant virus. One of these 'auto-excisable' systems consists of BACs flanked by cre recombinase target sites. For example, this system was used to co-infect Vero cells with the HSV-1 BAC and with an adenovirus vector that encodes the cre recombinase [71]. Another system utilizes endonuclease I-SceI cleavage and intramolecular Red recombination of inverted sequence duplications adjoining the prokaryotic vector backbone [34] and allows the markerless removal of all vector sequences upon virus reconstitution in eukaryotic cells. This system was applied to the analysis of an essential VZV tegument protein [72]. There are a number of reasons to think that these elegant experimental systems cannot be directly adapted to EBV BACs. For reasons previously stated, producer cell lines must be kept under antibiotic selection to avoid the rapid loss of the EBV episomes. Indeed, 293 cells are not dependent on EBV for growth and, although infected cells express EBNA1, they will lose episomes with time if the selection pressure is eased. Thus, it is currently impossible to excise the F-plasmid from EBV producer cells. It would therefore be necessary to activate the cre recombinase immediately before the onset of replication. This would require targeting every single replicating cell of the producer cell line, e.g. with an adenoviral vector carrying the cre recombinase, and then also require 100% efficiency of recombination and no interference with the replication process. Another drawback of this kind of strategy is that phenotypic markers such as GFP are lost in the process.

Another alternative would be to clone the BAC within EBV repeats, as recently suggested for rhadinoviruses [73]. Upon induction of lytic replication, the BAC backbone is eliminated as a result of recombination between the two flanking terminal repeats.

Finally, and perhaps most importantly, it is necessary to improve the quality of producer cell lines, in terms of generation time, level of virus production, and stability of the viral genome. Transfection of EBV DNA into a large panel of cell lines might help in identifying cells that show a high degree of permissivity as was previously observed for HEK293 or 293T cells. Steady improvements in our knowledge of the mechanisms that negatively control lytic replication might also have the very prosaic benefit of potentiating virus production.

## Conclusions

BAC recombinant technology has opened completely new areas of research for herpesviruses in general, but the benefits were particularly tangible for the study of gammaherpesviruses whose natural tendency to enter latency renders the study of infection and lytic replication difficult. This system has proven highly versatile and has virtually no limitations in terms of the genetic manipulation that it enables. There are now several systems available and the technology is being used by a growing number of laboratories. Nevertheless, construction of recombinant viruses remains tedious and time consuming. In particular, construction of a good producer cell line sometimes requires screening a large number of clones. In addition, it remains essential to perform all the necessary controls, e.g. the construction of revertants, which can be more demanding than the generation of the mutant itself. Future developments, some of which are already emerging, include the development of cell lines that efficiently support EBV lytic replication and do not lose this ability over time, cloning of more EBV strains, e.g. a type 2 EBV strain, and the design of recombinants in which the BAC backbone is auto-excisable.

## Competing interests

The authors declare that they have no competing interests

## Authors' contributions

All authors were involved in literature research, figures design and writing of the paper.
